# Personality Traits, Strategies of Coping with Stress and Psychophysical Wellbeing of Surgical and Non-Surgical Doctors in Poland

**DOI:** 10.3390/ijerph19031646

**Published:** 2022-01-31

**Authors:** Ewa Marcisz-Dyla, Józefa Dąbek, Tomasz Irzyniec, Czesław Marcisz

**Affiliations:** 1 Faculty of Management, Psychology, Katowice Business University, 40-659 Katowice, Poland; 2 Department of Cardiology, School of Health Sciences, Medical University of Silesia, 40-635 Katowice, Poland; jdabek@sum.edu.pl; 3 Department of Health Promotion and Community Nursing, Faculty of Health Sciences, Medical University of Silesia, ul. Medyków 12, 40-752 Katowice, Poland; tirzyniec@sum.edu.pl; 4 Department of Gerontology and Geriatric Nursing, Faculty of Health Sciences, Medical University of Silesia, 40-635 Katowice, Poland; klinwewtychy@poczta.onet.pl

**Keywords:** personality traits, coping with stress, resilience, psychophysical wellbeing, medical specialization

## Abstract

The aim of the study was to determine the personality traits, strategies of coping with stress and psychophysical wellbeing of surgical and non-surgical Polish doctors. The study used the following questionnaires and scales: the Resiliency Assessment Scale, the Type D Personality Scale, the Framingham Type A Scale, the Mini COPE—Coping Inventory and the Wellbeing Scale. Doctors performing surgical specialties were characterized by a significantly higher level of resilience components, a more frequent occurrence of the type B behaviour pattern and less frequent type D personality than doctors performing non-surgical specializations. The Mini COPE point values were comparable between surgical and non-surgical physicians. The sense of psychological wellbeing was higher in surgical specialists. The higher the values of the Optimistic approach to life and the ability to mobilize oneself in difficult situations, the lower the values of the Turning to religion domain and the higher the values of the Denial domain correlated with the performance of surgical specialization. Men performing surgical specializations were more often optimistic and inclined to consume alcohol, while women with non-surgical specialization more often coped with stress by turning to religion. We conclude that the personality traits of Polish doctors vary depending on their specialization. Physicians’ coping strategies do not differ depending on their specialization. The sense of mental wellbeing is higher in surgical specialists compared to non-surgical specialists. An optimistic approach to life and the ability to mobilize oneself in difficult situations, as well as coping with stress by denial are associated with the surgical specialization. Men performing surgical specialties more often declare optimism and a tendency to cope with stress by consuming alcohol or psychoactive substances, while women who perform non-surgical specializations more often cope with stress by turning to religion. Psychological screening tests and appropriate training, taking into account medical specialization, could be one way of improving resilience and coping with stress among doctors.

## 1. Introduction

The medical profession involving constant contact with illness, suffering and death is associated with an intense experience of stress [1,2]. In particular, doctors who perform shift work in hospitals are obliged to constant, direct contact with the patient [3], feeling excessive workload, which often results from the fast pace of work combined with difficult requirements, the need to make critical decisions based on ambiguous information, high intensity and long working time, as well as insufficient support [4].

Chronic occupational stress was experienced by physicians regardless of their specialization [5]. The results of studies conducted among Indian physicians showed significant differences in the intensity of experienced occupational stress depending on their specialization. It turned out that paediatricians were the most stressed-out group of doctors, and their stress levels were higher than that of anaesthesiologists, gynaecologists and surgeons. The lowest level of stress was experienced by general practitioners [1]. In a study of Polish doctors of various specialties, non-surgical doctors showed a higher level of emotional exhaustion, and the level of their empathy was higher compared to surgical specialists [6].

The specificity of working in the medical profession differs depending on the place of employment (clinics or hospitals) and the medical specialization performed. These differences concern the scope of competences, professional and social prestige, as well as economic and working conditions. The choice of specialization, as well as the choice of the medical profession, is influenced by such psychological factors as: personality traits, predispositions and interests [7,8,9]. There are a number of studies on the relationship between personality traits and the choice of specialization. However, a review by Borges et al. [10] suggested that it is difficult to draw unambiguous conclusions about the relationship between personality traits and the choice of medical specialization due to the variety of tools used to measure personality. On the basis of other studies conducted using the Eysenck Personality Questionnaire—Revised [11] and the personality inventories in the Big Five model [12,13,14,15]; however, confirmation of the relationship of personality traits with the declaration of choice or the actual choice of medical specialization was demonstrated both in the group of medical students in training and practicing doctors. It was also noted that the personality traits of physicians may moderate the relationship between medical specialization and the sense of wellbeing at work [16]. Undoubtedly, the knowledge of the personality traits of doctors played an important role in planning their professional careers [12].

One of the important personal characteristics, acting as a buffer in difficult situations, is resilience described in terms of a psychological resource contributing to effective coping with stress [17]. Resilience can be understood in two ways—as a personality trait (resiliency) and a process (resilience). The trait of resiliency means the cognitive and emotional ability to flexibly adapt to difficult situations. Resilience in the sense of the process means the dynamic adaptation in the face of difficulties and is expressed through effective coping despite the confrontation with a threatening situation [18,19]. An extensive and in-depth study of resilience was presented in the review by Southwick et al. [20] on the basis of a panel discussion of eminent experts. In a cohort of doctors from Great Britain, it was observed that the resilience was lower than in the general population. However, in this cohort, hospital-based doctors scored higher for resilience than general practitioners [21]. Resilience has also been shown to be associated with less burnout in family physicians [22]. The academic physicians with high burnout reported lower levels of resilience than those who were not burnt-out [23]. It was noticed that people with a higher level of resilience can be contrasted with people with type D personality traits [18,19], which may cause negative health consequences in terms of mental health disorders and lead to occupational burnout [24]. Close to the characteristics of the type D personality is the tendency to worry and the dominance of negative emotions that constitute neuroticism [14]. Type D personality might be a personality-related risk factor for burnout among emergency physicians [25].

Occupational stress of doctors is a common problem and coping with it can be understood as a multidimensional phenomenon, primarily serving the individual’s adaptation to stressful situations. Gastroenterologists have been shown to use both problem-focused and emotion-focused strategies [26]. Problem-focused coping is a potentially more adaptive coping strategy in gastroenterology practice and may explain lower levels of reported burnout, distress, and increased job-related self-efficacy. A study of Pakistani gynecologists showed more frequent use of problem-focused stress coping strategies, in particular, such as active coping, planning, acceptance and positive reframing, as well as the use of instrumental social support [27]. Another study found that most physicians used adaptive coping strategies; however, maladaptive strategies (alcohol/drugs, denial, disengagement) were positively associated with anxiety, depression and stress [28]. Doctors from the United Kingdom most often used maladaptive coping strategies [21].

Defining the psychological characteristics of doctors overloaded with work in the surgical or non-surgical specialty may be useful for learning and implementing the methods of shaping the psychological profile aimed at improving the satisfaction and quality of the profession.

The aim of this study was to evaluate the personality traits, strategies of coping with stress and psychophysical wellbeing of doctors performing surgical and non-surgical specialties in Poland, as well as to determine the relationships between the above-mentioned psychological indicators and the medical specialization performed. 

## 2. Materials and Methods

### 2.1. The Subjects

The study was conducted in 655 doctors, including 401 women and 254 men. In the examined group, 266 doctors (134 women and 132 men) performed surgical specializations, and 389 (267 women and 122 men) non-surgical specializations. The surveyed doctors had medical specialization or were in the process of implementing it. The surgical specializations included: anaesthesiology and intensive therapy, general surgery, pediatric surgery, vascular surgery, plastic surgery, gynaecology and obstetrics, invasive cardiology, neurosurgery, ophthalmology, orthopaedics and traumatology, otolaryngology, and urology, whereas non-surgical specializations were as follows: allergology, lung diseases, internal diseases, dermatology, diabetology, endocrinology, gastroenterology, geriatrics, hematology, cardiology, nephrology, neonatology, neurology, oncology, pathomorphology, paediatrics, psychiatry, radiology, oncological radiotherapy, medical rehabilitation and rheumatology. The age of the subjects ranged from 26 to 75 years; the mean age was 38.9 ± 11 (mean ± SD) years. The mean age of surgical physicians was 37.9 ± 10.3 years, and of non-surgical physicians—39.6 ± 11.4 years. The job seniority ranged from 2 to 51 years; the mean job seniority was 12.9 ± 11 years. Doctors of surgical and non-surgical specializations were statistically comparable in terms of age and job seniority (*p* > 0.05). 

The study involved Polish doctors employed in 30 randomly selected hospitals in the Silesian Voivodeship after obtaining the consent of the management of hospitals to conduct the survey. A total of 780 sets of questionnaires were handed out to doctors who sequentially randomly agreed to complete them. In total, 712 sets were returned, which accounted for 91.3% of the sets distributed. Overall, 57 sets were excluded, because they were incomplete. Answering the questions included in the set of prepared questionnaires were voluntary and anonymous. The study was carried out in 2017–2018. From the calculation of the representativeness of the sample at the confidence level of 95% in the studied group of 655 doctors, the estimation error was only 3.73%, which meant that the studied group was representative. The inclusion criteria for the study were: being professionally active, having at least 2 years of experience in the medical profession, consent to participate in the study and filling out all the received questionnaires.

The Bioethics Commission of the Medical University of Silesia in Katowice decided that the survey nature of the study does not require its consent to conduct the research (KNW/0022/KB/190/17).

### 2.2. Methods

The study used scales and questionnaires to measure psychological variables, such as: the Resiliency Assessment Scale—SPP 25, the Type D Personality Scale—DS 14, the Framingham Type A Scale, the Mini COPE—Coping Inventory and The Wellbeing Scale from the Psychosocial Working Conditions questionnaire.

#### 2.2.1. The Resiliency Assessment Scale—SPP-25 by Ogińska-Bulik and Juczyński

The scale is used to measure the overall score of resilience level, as well as five factors of resilience, namely: (1) Persistence and determination in action, (2) Openness to experience and sense of humour, (3) Individual’s ability to cope and tolerance of negative emotions, (4) Tolerance of failure and viewing life as a challenge, and (5) An optimistic approach to life and the ability to mobilize oneself in difficult situations. The respondents marked the answers on a 5-point scale: ‘definitely not’ (0 points), ‘rather not’ (1 point), ‘hard to say’ (2 points), ‘rather yes’ (3 points), and ‘definitely yes’ (4 points). A higher score in the questionnaire indicated a greater intensity of resilience and its particular indicators. The overall score of resilience can be expressed on the sten scale, using the following interpretation: 1–4 sten—low resilience, 5–6 sten—average resilience, 7–10 sten—high resilience. The reliability of the tool, measured with the Cronbach’s alpha coefficient, was 0.89 for the entire scale, while the reliability of the 5 subscales ranged from 0.67 to 0.75 [18].

#### 2.2.2. The Type D Personality Scale—DS 14 by Ogińska-Bulik, Juczyński and Denolett

The scale is used to measure the intensity of the Type D personality, which is a so-called distressed personality. It is a scale consisting of 14 statements, measuring the general Type D personality indicator along with its dimensions, defining negative affectivity (a tendency to experience negative emotions) and social inhibition (conscious refraining from revealing negative emotions in contacts with other people for fear of social rejection). The examined doctors responded to the statements using a 5-point scale, namely: 0—false, 1—rather false, 2—difficult to say, 3—rather true, 4—true. The presence of a Type D personality is confirmed by a score of ≥10 points for both dimensions of Type D personality. Cronbach’s alpha coefficient for the negative emotionality scale was 0.86, and for social inhibition—0.84 [29].

#### 2.2.3. The Framingham Type A Scale in the Polish Adaptation of Juczyński 

The scale is used to measure the Type A behaviour pattern. It consists of 10 statements concerning the features and properties that describe the individual (the first 5 statements), the feelings experienced by the individual at the end of work or homework (4 subsequent statements) and time pressure (the last statement). The scale measures the two main indicators of Type A behaviour, namely, hassle and rivalry. Responses to the first five statements are assigned appropriate weights: definitely (1), probably (0.67), probably not (0.33), and definitely not (0). The next five items contain two answer options: yes (1), no (0). The total score is the mean of all scores; the scores for each factor are the mean of the answers to the questions for each factor. The overall result as well as the results of both factors range from 0 to 1. If the value is closer to 1, it indicates the dominance of Type A behaviour, which means a high tendency to compete and a sense of time pressure, while the result closer to 0 suggests the dominance of Type B behaviour, indicating a low tendency to compete and no time pressure. The scale result may be useful in assessing the determinants of cardiovascular diseases and other somatic diseases. The reliability of the scale, measured by the Cronbach’s alpha coefficient, was 0.62 [30].

#### 2.2.4. The Mini COPE—Coping Inventory by Carver, in the Polish Adaptation of Juczyński and Ogińska-Bulik

The tool, which is the shortened version of a multi-dimensional inventory for measuring coping with stress—the COPE Inventory (The Coping Orientation to Problems Experienced) was used to evaluate the applied coping strategies. It consists of 28 statements covering 14 strategies of coping with stress, namely: Active Coping, Planning, Positive Reinterpretation, Acceptance, Humour, Turning to Religion, Seeking of Instrumental Social Support, Seeking of Emotional Social Support, Mental Disengagement, Denial, Focus on and Venting of Emotions, Substance Use, Behavioural Disengagement and Self-Blame. Each strategy is contained in two statements. When completing the questionnaire, one answer out of four is selected: ‘I almost never do this’ (0 points), ‘I rarely do this’ (1 point), ‘I do this often’ (2 points) or ‘I almost always do this’ (3 points). The higher the score, the greater the intensity of using a given strategy [31]. Among the above-mentioned methods of coping with stress, two separate groups of problem-focused strategies (Active Coping, Planning, Seeking of Instrumental Social Support) and emotional behaviours (Turning to Religion, Seeking of Emotional Social Support, Denial) were distinguished. The half-reliability for 14 scales measured by the Spearman and Brown’s half-reliability coefficient was 0.86 [32].

#### 2.2.5. The Wellbeing Scale from the Psychosocial Working Conditions Questionnaire

The Wellbeing Scale is used to measure the level of perceived psychophysical wellbeing, divided into two factors: Physical wellbeing and Mental wellbeing. The Wellbeing Scale consists of 22 questions. Below each of the questions there were 5 possible answers, of which one should be marked, scored from 1 to 5. The higher the total score, the greater the sense of wellbeing. The questionnaire possessed good psychometric properties; the reliability for the scales measured with the Cronbach’s alpha coefficient ranged from 0.82 to 0.94 [33].

### 2.3. Statistical Analysis

The obtained results were analyzed with the SPSS 24 statistical calculation program. The descriptive statistics of quantitative variables were taken into account, with the normality of their distributions checked using the Shapiro-Wilk test. It was shown that the measured variables differed statistically with their distribution from the normal distribution; therefore, non-parametric tests were used in the further part of the analysis. The Cronbach’s alpha coefficient was determined for each of the scales used in the study. The subjects were classified into the appropriate type A/B and intermediate behaviours, taking into account their responses in the Framingham Type A Scale; they were also divided into type D and non-D personality based on the responses obtained in the Type D Personality Scale. In order to determine the relationship between nominal variables and the specializations performed by the subjects, the chi-square test of independence was used. In order to verify statistically significant differences between doctors working in surgical and non-surgical wards, the statistics were calculated using the Mann–Whitney U test. Logistic regression models were developed, in which surgical or non-surgical specialization was assumed as a dependent variable. The statistical significance was assumed at the level of *p* ≤ 0.05.

## 3. Results

Men, more often than women, chose surgical specializations, while women chose non-surgical specializations (*p* < 0.01). The marital status of the subjects did not significantly differentiate them in terms of the selected surgical or non-surgical specializations (*p* = 0.492) (Table 1).

### 3.1. Personality Traits

In comparison with the doctors performing non-surgical specializations, surgical doctors were characterized by statistically significantly higher scores in terms of the overall dimension of resilience and the following components: Individual’s ability to cope and tolerance of negative emotions, Tolerance to failure and viewing life as a challenge, An optimistic attitude to life and the ability to mobilize oneself in difficult situations (*p* < 0.01) (Table 2). Cronbach’s alpha values, determining the internal consistency of the components of the scale, were 0.62–0.93.

In non-surgical physicians, the level of Negative affectivity and the total score of the Type D Personality Scale were higher than in surgical physicians (*p* < 0.05–0.01) (Table 2). Cronbach’s alpha values for the components of the scale were 0.83–0.89.

In doctors with non-surgical specializations, the intermediate behaviour pattern was the most common (40.6%); the least frequent was type B (25.7%). The frequencies of all behaviour patterns, i.e., A, intermediate, and B types were comparable in surgical specialists. The differentiation in the occurrence of behavioural patterns, especially type B, between the groups of surgical and non-surgical physicians turned out to be statistically significant (*p* < 0.05) (Table 2). The Cronbach alpha value of the scale was 0.62.

### 3.2. Coping with Stress

The point values of almost all Mini COPE components did not differ significantly between the two groups (*p* > 0.05), with the exception of the Turning to religion domain with a higher point value in non-surgical physicians (*p* = 0.001) (Table 3). Due to the low reliability of the Cronbach’s alpha coefficient in the two domains of the Mini COPE scale, i.e., Mental Disengagement and Focus on and Venting of Emotions, they were excluded from the statistical analysis. The values of Cronbach’s alpha coefficients for the remaining domains of the scale were 0.63–0.9.

### 3.3. Psychophysical Wellbeing

The point values for Physical Wellbeing and General Wellbeing were statistically comparable in surgical and non-surgical physicians (*p* > 0.05), while the level of Mental Wellbeing was higher in surgical physicians (*p* = 0.027) (Table 4). Cronbach’s alpha values for the components of the scale were 0.81–0.89.

### 3.4. Independent Variables Explaining the Type of Specialization

The statistical calculations included logistic regression models, in which the dependent variable was the medical surgical or non-surgical specialization and the independent variables were: the level of resilience and its dimensions, the A/B behaviour pattern, type D personality, coping strategies and psychophysical wellbeing.

The first logistic regression model showing the independent variables explaining the choice of medical specialization in the examined group of physicians explains 8.2% of the variability. However, it can be concluded that the higher the values of the Optimistic approach to life and the ability to mobilize oneself in difficult situations (B = −0.105; *p* < 0.001), the lower the values of the Turning to religion domain (B = 0.163; *p* < 0.001) and the higher the values of the Denial domain (B = −0.171; *p* = 0.004), the greater the likelihood of doctors choosing a surgical specialization (Table 5).

The second logistic regression model showing the independent variables explaining the choice of medical specialization in the examined group of male physicians explains 9.4% of the variability. The choice of surgical specialization by men correlated with higher values of the subscale: An optimistic approach to life and the ability to mobilize oneself in difficult situations (B = −0.131; *p* = 0.002) and in those men who were more likely to consume alcohol or take other psychoactive substances (B = −0.324; *p* = 0.002) (Table 5).

The third logistic regression model showing the independent variables explaining the choice of medical specialization in the examined female doctors explains 2.9% of the variability. The choice of surgical specialization by women turned out to be co-variable with lower values in the domain of Turning to religion (B = 0.163; *p* = 0.004) (Table 5).

## 4. Discussion

This study included Polish professionally-active doctors, women and men, specializing in both surgical and non-surgical specialties. The specialization was considered the main criterion differentiating the studied group and, on this basis, subjects performing surgical and non-surgical specializations were compared in terms of psychological variables—personality traits, coping strategies and psychophysical wellbeing. In line with the objectives set out in the study, the analysis covered the occurrence of the type A/B behaviour pattern and type D personality in the examined group, which could contribute to the development of psychosomatic symptoms. The occurrence of a personality property called resilience, supporting effective activities in the face of stress, was also analyzed.

It was shown that doctors performing surgical specialities were characterized by a greater intensity of resilience, including its components, such as the individual’s ability to cope and tolerance of negative emotions, tolerance of failure and viewing life as a challenge and an optimistic approach to life and the ability to mobilize oneself in difficult situations than non-surgical doctors. Resilience was described in terms of a psychological resource contributing to effective coping with stress [17]. This feature means the process of dynamic adaptation in the face of difficulties and is expressed through effective coping despite confronting the threatening situation [18,19]. Therefore, it can be assumed that, in the case of the surgical doctors we studied, the effectiveness of coping in a stressful situation was higher than in the case of non-surgical doctors. Moreover, in the study of the United Kingdom doctors, a higher value of resilience score was observed in surgical specialties than in non-surgical specialties [21]. In a study of American physicians across specialties, resilience scores were highest in doctors specialized in emergency medicine, neurosurgery, and preventive and occupational medicine, and they were lowest in general paediatricians, neurologists and gynaecologists [34]. The level of resilience determined using the Connor Davidson Resilience Scale was inversely associated with burnout symptoms [34]. In addition, in another study among resident physicians, high resilience was associated with a lower risk of burnout [35]. According to Card [36], effective training in developing resilience as the ability to adapt successfully and favour well-being can have many benefits, especially when a practitioner is dealing with the difficult emotions associated with the inevitable suffering and death of patients. The observed differences in resilience across specialties and the association of resilience with burnout within each specialty are intriguing and merit further study. 

In the conducted study, it was shown that non-surgical physicians experienced a greater intensity of type D personality traits than surgical physicians, and non-surgical physicians experienced negative emotions more often than surgical specialists. Type D distressed personality affects the perception of work as stressful, leading to job burnout and causing negative health consequences, e.g., in the form of deterioration of mental health [24]. Based on our research showing a lower level of resilience and a greater intensity of type D personality in physicians performing non-surgical specializations, it can be concluded that they were more exposed to the risk of occupational burnout. This link may be complemented by the observation in our study that the occurrence of type B personality is less frequent in the group of non-surgical physicians than in the group of surgical physicians, which suggests that non-surgical physicians were characterized by a lower level of relaxation and focus on quality of life, as well as greater ambition and less patience. The indirect personality type was more common in non-surgical doctors than in surgical doctors. However, a study of Greek doctors showed that surgeons presented a significantly higher level of hostility, which is a component of the type A behaviour pattern, compared to the non-surgical specialists [37]. In a study comparing resident otolaryngologists and non-specialized doctors, a greater intensification of the features of type A behaviour pattern was observed in future otolaryngologists, which may also make them more susceptible to experiencing job burnout [38].

A higher level of psychological wellbeing has been shown in the examined surgical doctors compared to non-surgical doctors. The results in the field of mental wellbeing of surgical doctors were inconsistent with the results of studies conducted among Polish doctors of surgical and non-surgical specializations [39], which proved that surgeons and emergency medicine doctors experienced lower life satisfaction as opposed to paediatricians, who had the highest level of life satisfaction. The reasons for the worse wellbeing of emergency medicine physicians were considered to be the heavy workload resulting from the specificity of their work in the form of night shifts and participation in rescue operations. The examined surgeons seemed to experience more frequent negative emotions [39]. Back et al. [40] suggested that the wellbeing of palliative care physicians is influenced by both the requirements related to the specificity of work and personal resources in the form of resilience, which should be developed by training various skills, e.g., learning emotional self-regulation or recognizing cognitive distortions. A study conducted in a group of healthcare professionals (including doctors) showed that employees who were less tired, more emotionally involved and experienced a greater sense of physical wellbeing were more sensitive to the needs of patients and their families, and maintained better contacts with colleagues, and the quality of their care was higher [41].

In this study with the use of logistic regression, it was shown that surgical specializations were more often performed by doctors with an optimistic attitude to life and with the ability to mobilize themselves in difficult situations, more often using denial in stressful situations, and men more often using alcohol or other stimulants. In the study by Basińska and Dziewiątkowska [42], it was also observed that positive thinking and direct action were the characteristic strategies of coping with stress used by surgeons. Moreover, it was noticed that, in surgeons with longer work experience, avoidance strategies appeared more often [42], and the results of our study confirmed that surgical specialists used such strategies as: denial, which is an example of avoiding confrontation with a difficult situation, and the use of alcohol. Denial is a strategy of ignoring a stressful situation that, in the long run, can be classified as a maladaptive coping strategy. The study by Wallace and Lemaire [43] showed that doctors who used denial in stressful situations more often experienced a sense of occupational burnout in the form of emotional exhaustion. On the other hand, it was noticed that positive emotionality understood as optimism acted as a buffer protecting doctors against the negative effects of the denial strategy by reducing the probability of occupational burnout [43]. Presumably, a similar tendency occurred among the examined group of surgical specialists. On the other hand, non-surgical specializations were more often performed by doctors heading towards religion in a stressful situation; this concerned the entire group of examined doctors and, separately, the women themselves. Turning to religion is a strategy that can provide emotional support and help to positively re-evaluate a stressful situation. Over time, a strategy involving the use of alcohol and other stimulants was distinguished, which was previously treated as an example of a distraction strategy [44]. Doctors experience many occupational stressors, so they use various coping strategies to deal with them. The studies by King et al. [45] and Lemaire and Wallace [46] showed that the most common strategies of coping with stress were: active coping and planning, and the rarest—distracting attention from stress by focusing on alternative activities, and other escape strategies.

In order to improve the level of resilience, coping with stress and reducing the risk of job burnout in doctors, the following strategies could be implemented: psychological screening of residents in terms of their personality traits and mental health characteristics, taking into account the choice of medical, surgical or non-surgical specialization; support in the development of training various skills to increase the level of resilience, especially in non-surgical doctors (training in resilience should be a preventive strategy); training in stress management aimed at eliminating some phenomena related to burnout; integrating emotional intelligence education into medical students’ curricula for a burnout buffering effect.

### Strengths and Limitation

One of the strengths of the study is its anonymity, as it was considered a sufficient incentive to participate in the study and to give honest responses to the questions concerning such a sensitive area as individual experiences and behaviours. A high response rate was obtained (80%), and the study group turned out to be numerous; it was a representative sample for the population of doctors in Silesia working in a dozen or so hospitals, which makes it possible to generalize the obtained results. The study used standardized scales and questionnaires with high or medium reliability. On the other hand, due to the self-report character, the study is not devoid of certain limitations, namely, that the effect of social expectations cannot be ruled out, i.e., the tendency of respondents to provide socially acceptable answers, not necessarily consistent with the facts. Another limitation of the study was the more static approach to the analyzed indicators of resilience and coping with stress, thus, restricting interpretation possibilities.

## 5. Conclusions

The personality traits of doctors varied depending on the performed specialization: the level of resilience turned out to be higher in doctors performing surgical specializations; the type B behaviour pattern was also more common in surgical doctors, while the type D personality, especially a tendency to negative affectivity, was found more often in doctors performing non-surgical specializations. Doctors’ coping strategies do not differ depending on the specialization. The sense of mental wellbeing was higher in surgical specialists compared to non-surgical specialists. An optimistic approach to life and the ability to mobilize oneself in difficult situations, as well as coping with stress by denial, were related to the performance of a surgical specialization. Men performing surgical specialties more often declared optimism and a tendency to cope with stress by consuming alcohol or psychoactive substances, while doctors, especially women who chose non-surgical specializations, more often coped with stress by turning to religion. Psychological screening tests and appropriate training, taking into account medical specialization, could be one way of improving resilience and coping with stress among doctors.

## Figures and Tables

**Table 1:** Gender and marital status of the examined group of doctors and the type of specialization.

Parameter		Examined Group of Doctors (*n* = 655)			
		Specialization		Statistical Significance of the Differences	
		Surgical (*n* = 266)	Non-Surgical (*n* = 389)	Chi^2^	*p*
Gender (*n*/%)	males	132/52.0	122/48.0	22.191	<0.01
	females	134/33.4	267/66.6		
Marital status (*n*/%)	married	167/38.6	266/61.4	2.407	0.492
	single	81/45.3	98/54.7		
	divorced	14/41.2	20/58.8		
	widowed	4/44.4	5/55.6		

*n* = number; *p* = statistical significance of the difference.

**Table 2:** The level of resilience, type D personality and patterns of behaviour of the examined group of doctors, taking into account specializations.

Scale	Components	Examined Group of Doctors (*n* = 655)			
		Specialization		*p* U Mann-Whitney Test	
		Surgical (*n* = 266)	Non-Surgical (*n* = 389)		
		Mean (SD); Median; Range	Mean (SD); Median; Range		
The Resiliency Assessment Scale (in points) Mean (SD); median; range	Persistence and determination in action	14.76 (3.28); 15; 3–20	14.39 (2.99); 14; 3–20	0.059	
	Openness to experience and sense of humour	15.52 (2.77); 16; 7–20	15.14 (2.66); 15; 5–20	0.072	
	Individual’s ability to cope and tolerance of negative emotions	14.58 (3.28); 15; 4–20	13.70 (3.18); 14; 6–20	<0.01	
	Tolerance of failure and viewing life as a challenge	14.82 (3.33); 15; 4–20	13.94 (2.99); 14; 5–20	<0.01	
	An optimistic approach to life and the ability to mobilize oneself in difficult situations	13.85 (3.40); 14; 4–20	12.80 (3.34); 13; 3–20	<0.01	
	Resilience—sum	75.52 (13.84); 75; 31–100	69.97 (13.14); 69; 36–100	<0.01	
The Type D Personality Scale (in points) Mean (SD); median; range	Negative affectivity	10.99 (6.13); 11; 0–28	12.23 (5.70); 12; 0–27	0.006	
	Social inhibition	10.06 (5.88); 10; 0–27	10.69 (5.69); 11; 0–27	0.139	
	Personality D—sum	21.05 (10.58); 20; 0–55	22.92 (9.72); 22; 0–48	0.016	
Patterns of behaviour according to the Framingham Type A Scale (*n*/%)	Type A	88/33.1	131/33.7	Chi^2^ = 6.002	<0.05
	Intermediate type	88/33.1	158/40.6		
	Type B	90/33.8	100/25.7		

*n* = number; SD = standard deviation; *p* = statistical significance of the difference.

**Table 3:** Coping strategies in the examined group of doctors, taking into account specializations.

Coping Strategies (in Points)	Examined Group of Doctors (*n* = 655)		
	Specialization		*p* U Mann-Whitney Test
	Surgical (*n* = 266)	Non-Surgical (*n* = 389)	
	Mean (SD); Median; Range	Mean (SD); Median; Range	
Active Coping	4.57 (1.15); 5; 0–6	4.52 (1.10); 4; 1–6	0.385
Planning	4.48 (1.11); 4; 0–6	4.54 (1.13); 4; 1–6	0.632
Positive Reinterpretation	3.76 (1.26); 4; 0–6	3.60 (1.37); 4; 0–6	0.099
Acceptance	3.83 (1.20); 5; 0–6	3.87 (1.13); 4; 1–6	0.889
Humour	2.17(1.39); 2; 0–6	2.02 (1.33); 2; 0–6	0.180
Turning to Religion	1.92 (1.84); 2; 0–6	2.44 (1.93); 2; 0–6	0.001
Seeking of Emotional Social Support	3.70 (1.55); 4; 0–6	3.90 (1.57); 4; 0–6	0.091
Seeking of Instrumental Social Support	3.76 (1.48); 4; 0–6	3.91 (1.44); 4; 0–6	0.227
Denial	1.58 (1.49); 1; 0–6	1.35 (1.30); 1; 0–6	0.095
Substance Use	1.00 (1.45); 0; 0–6	0.80 (1.32); 0; 0–6	0.083
Behavioural Disengagement	1.42 (1.25); 1; 0–5	1.40 (1.21); 1; 0–6	0.969
Self-Blame	2.55 (1.66); 2; 0–6	2.75 (1.48); 3; 0–6	0.081
Problem-focused Strategies	4.27 (0.91); 4.33; 0–6	4.33 (0.95); 4.33; 1–6	0.799
Emotional Behaviours	2.40 (1.05); 2.33; 0–5.67	2.56 (1.03); 2.67; 0–6	0.074

*n* = number; SD = standard deviation; *p* = statistical significance of the difference.

**Table 4:** The level of psychophysical well-being of the examined group of doctors, taking into account specializations.

The Well-Being Scale (in Points)	Examined Group of Doctors (*n* = 655)		
	Specialization		*p* U Mann-Whitney Test
	Surgical (*n* = 266)	Non-Surgical (*n* = 389)	
	Mean (SD); Median; Range	Mean (SD); Median; Range	
Physical well-being	3.92 (0.53); 3.96; 2.45–5.00	3.88 (0.57); 4.00; 2.27–4.91	0.692
Mental well-being	3.53 (0.57); 3.55; 1.55–5.00	3.44 (0.52); 3.45; 1.64–5.00	0.027
General well-being	3.72 (0.49); 3.73; 2.36–5.00	3.66 (0.49); 3.68; 2.27–4.86	0.142

*n* = number; SD = standard deviation; *p* = statistical significance of the difference.

**Table 5:** Logistic regression for independent variables explaining the choice of specialization in the examined group of doctors.

Group of Doctors	Independent Variable	Specialization			
		B	*p*	Exp (B)	Likelihood Logarithm/R^2^ by Nagelkerke
All (*n* = 655)	An optimistic approach to life and the ability to mobilize oneself in difficult situations	−0.105	<0.001	0.900	843.829/0.082
	Turning to Religion	0.163	<0.001	1.177	
	Denial	−0.171	0.004	0.843	
	Constant	2.123	<0.001	8.354	
Males (*n* = 254)	An optimistic approach to life and the ability to mobilize oneself in difficult situations	−0.131	0.002	0.878	333.181/0.094
	Substance Use	−0.324	0.002	0.723	
	Constant	2.093	0.001	8.108	
Females (*n* = 401)	Turning to Religion	0.163	0.004	1.177	502.511/0.029
	Constant	0.317	0.054	1.372	

B = coefficient of logistic regression function, *p* = statistical significance, Exp (B) = odds ratio.

## Data Availability

The data could be obtained from the corresponding author on reasonable request.
